# Case of Purpura Hæmorrhagica

**Published:** 1845-07

**Authors:** John Barclay

**Affiliations:** Leicester


*Case of Purpura Hæmorrhagica*, by John Barclay, M. D., of Leicester.—On the 23rd of last April I saw, accidentally, with Mr. Buck, (who kindly peimits the publication of the following notes,) a Union patient, to whom he had just been summoned.

Mary Johnston, aged 11, a fair and stout child, living in a low situation, and in a neighborhood where some cases of small pox, and a good many of the worst form of typhus, were then occurring. She had been ailing for four or five weeks, and had kept her bed for ten days, having been prescribed for by an irregular practitioner. She had complained of headache and thirst; and the bowels had been opened by medicine. About one, p. m., of the preceding day, (Tuesday,) she began to bleed at the nose and lips; and on Wednesday morning vomited a quantity of dark blood four or five times. (The mother states there was about a teacupful each time.) She had also had three copious and most offensive stools that morning, consisting of black clotted blood.

At eleven, a. m., we found the patient pale, but with a flush on the cheek; skin very hot and harsh; breathing rapid; pulse 140, small and compressible; the tongue bloodless, and furred at the back part; and the abdomen tympanitic. The blood was draining down in a small continuous stream from the nostrils, as she lay on her back; and on the arms, thighs, and abdomen, were many small spots of purpura, very beautifully marked, the largest being about two lines in diameter; on one arm was a large vibex. Some wine was administered immediately, and Mr. Buck agreed to try the following:—To take a powder of gallic acid of five grains, white sugar ten grains, every four hours, and a stimulant mixture, with carbonate of ammonia.

About one p. m., the bleeding at the nose ceased, after lasting twenty-four hours. At five she had another stool of the same nature as the previous ones. The third and fourth powders produced sickness, and only her drinks, without any blood, were rejected.

On Thursday she continued much in the same state. She was directed to continue the powders, which had been temporarily suspended on account of the vomiting; and a sinapism was applied to the abdomen.

Friday, 25th. She had taken one drachm of the gallic acid, and seemed decidedly better. The pulse was 120; the tongue covered with a brown fur, and the abdomen was still tympanitic. The spots continued as brilliant as before, and her arm showed a very marked discolouration at a part which had been purposely pinched the day before. She was directed to omit the powders and continue the stimulant mixture.

From this time she went on improving; the spots gradually lost their brilliancy, and disappeared of a light brown colour, being still perceptible ten days after her seizure, when she was up, and taking tonics and light nourishment. The bowels and skin acted very freely after the first week, and she is now, (May 4th,) quite well.—***Prov. Med. and Sur. Journ.***

